# Trapping of drops by wetting defects

**DOI:** 10.1038/ncomms4559

**Published:** 2014-04-11

**Authors:** Dieter 't Mannetje, Somnath Ghosh, Rudy Lagraauw, Simon Otten, Arjen Pit, Christian Berendsen, Jos Zeegers, Dirk van den Ende, Frieder Mugele

**Affiliations:** 1 University of Twente, MESA+ Institute for Nanotechnology, Physics of Complex Fluids, PO Box 217, 7500 AE Enschede, The Netherlands; 2 Department of Applied Physics, Mesoscopic Transport Properties Group, Eindhoven University of Technology, P.O. Box 513, 5600 MB Eindhoven, The Netherlands

## Abstract

Controlling the motion of drops on solid surfaces is crucial in many natural phenomena and technological processes including the collection and removal of rain drops, cleaning technology and heat exchangers. Topographic and chemical heterogeneities on solid surfaces give rise to pinning forces that can capture and steer drops in desired directions. Here we determine general physical conditions required for capturing sliding drops on an inclined plane that is equipped with electrically tunable wetting defects. By mapping the drop dynamics on the one-dimensional motion of a point mass, we demonstrate that the trapping process is controlled by two dimensionless parameters, the trapping strength measured in units of the driving force and the ratio between a viscous and an inertial time scale. Complementary experiments involving superhydrophobic surfaces with wetting defects demonstrate the general applicability of the concept. Moreover, we show that electrically tunable defects can be used to guide sliding drops along actively switchable tracks—with potential applications in microfluidics.

## Abstract (web summary)

Precise control of moving droplets on surface defects is essential for microfluidic applications. Here the authors formalize a generic rule to describe droplet trapping using two dimensionless parameters and show how electrically tunable defects guide sliding drops in microfluidic channels.

## Graphical abstract

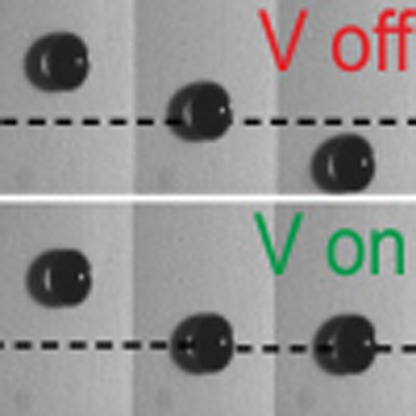

Drops moving along solid surfaces are ubiquitous. We encounter them on rainy days on the windows of our houses, cars, trains and airplanes and in many technological applications including cleaning and coating technology and two-phase flow microfluidics^1^,^2^,^3^. Wetting defects such as topographic patterns and chemical patches of variable wettability can trap drops, prevent their removal or allow for steering them in certain directions with important consequences for the efficiency of drop condensation from vapour in heat exchangers and fog harvesters^4^,^5^,^6^. Animals^7^ and plants^8^ in arid environments make use of specific wettability patterns to collect humidity more efficiently. Conversely, insects^9^ and plants^10^ in humid environments frequently make use of anisotropic wetting structures to steer impinging rain drops off their surface. While the critical conditions governing the detachment of pinned drops from wetting defects have been addressed in considerable detail^11^,^12^,^13^,^14^,^15^, the physical parameters controlling the inverse process, namely the capture and guidance of mobile drops by wetting defects, is largely unexplored. From a purely static perspective, one would expect that both processes are governed by the same local force balance at the contact line that has been studied extensively^11^,^12^,^16^ following the seminal work by de Gennes and coworkers^17^,^18^ on the wetting of heterogeneous surfaces in the 1980s. For moving drops, however, dynamic effects can lead to richer dynamics than those dictated by the static energy landscape due to surface heterogeneity. The most generic question that arises is whether or not a certain wetting defect is strong enough to capture a passing drop. As we will show in this work, the answer to this question involves not only the pinning forces exerted by the defect but also the balance between inertia and viscous dissipation of the drop. For aqueous drops of millimetric size and common wetting defects, all three forces turn out to be of the same order of magnitude. This results in a rather sharp transition between the extreme cases of overdamped dynamics for more viscous liquids and very weakly damped situations, such as Leidenfrost drops, that lose little energy upon passing a single defect^19^,^20^.

In this work, we determine the critical conditions for drop trapping over a wide range of driving forces, defect strengths and drop properties (size, viscosity). To vary the driving force, we change the slope angle of an inclined plane. To vary the defect strength, we make use of recently introduced electrically tunable wetting defects based on electrowetting^21^, as well as topographic and chemical defects on superhydrophobic surfaces. The former approach enables a continuous variation of the energy landscape experienced by the drop upon tuning the externally applied voltage while leaving the chemical and topographical homogeneity of the surface intact.

## Results

### Inclined plane setup

**Figure 1:**
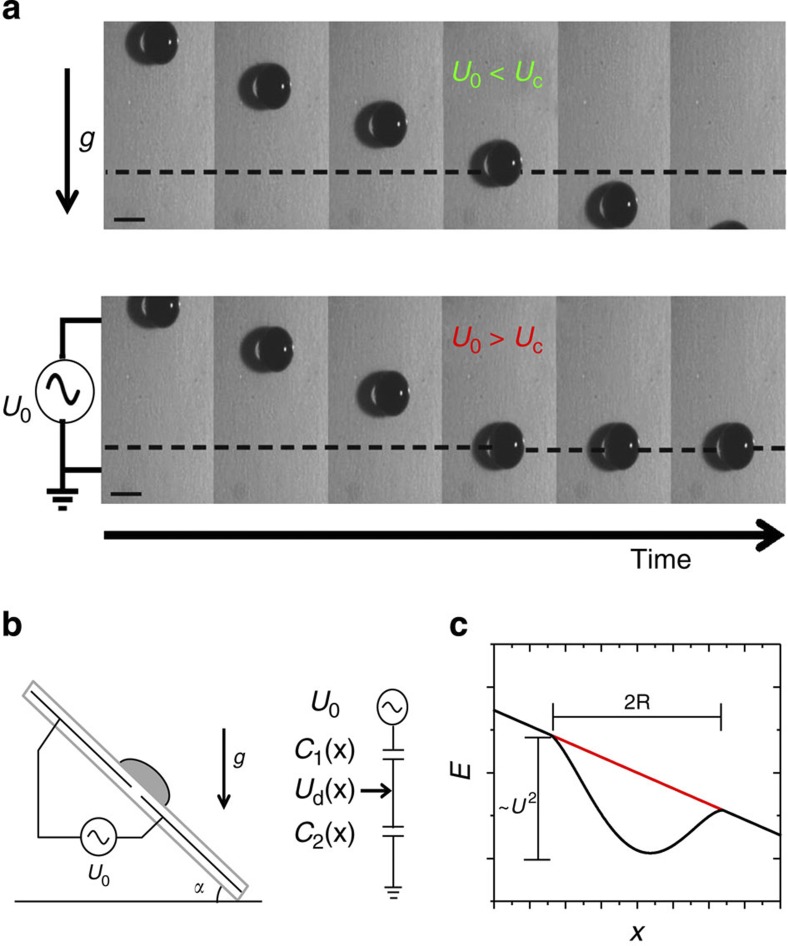
Drop trapping at electrically tunable wetting defect. (**a**) Snapshots of sliding drop (volume: 60 μl; inclination angle: 4.3°). Top: applied voltage *U*_0_=200 V smaller than the critical trapping voltage *U*_C_. Bottom: *U*_0_=400 V, larger than *U*_C_. (see also Supplementary movie 1). (**b**) Schematic view of the setup illustrating inclined plane and the electrodes forming the electrical trap along with equivalent electrical circuit diagram (see Methods section for details). (**c**) Schematic view of the potential energy landscape versus drop position for zero voltage (red line) and a finite voltage U (black) according to equation 1. *U*_d_ is the potential of the drop.

We consider a millimetre-sized drop sliding down an inclined plane. Unless stated otherwise, the substrate surface consists of a thin oil-impregnated polymer film with a Young contact angle *θ*_Y_ of ~90° and a very small contact angle hysteresis Δ*θ*<3° resulting in a very small roll-off angle just below *α*_0_≈3°. Inclination angles of *α*=3...15° give rise to steady sliding speeds *v*_0_ ranging from a few mm s^−1^ to several cm s^−1^. This corresponds to capillary numbers of *Ca*=*μv*/*γ*≈10^−4^...10^−3^, where *μ* is the viscosity and *γ* the surface tension. As viscous forces are small compared to capillary forces, the drops retain their essentially half-spherical shape during sliding. On the lower part of the surface, there is a horizontal wetting defect oriented perpendicular to the trajectory of the drop, as indicated by the dashed lines in Fig. 1a. In practice, the defect consists of two electrodes submerged below the polymer film that are separated by a small gap (see Methods for details).

### Interaction between drops and electrical traps

Figure 1 shows a series of snapshots of two drops sliding down the inclined plane for two different voltages. Above the defect, both drops assume a constant steady sliding velocity *v*_0_ that is determined by the balance of the (effective) gravitational driving force and frictional (viscous) dissipation. Upon reaching the defect, the drop gets trapped if the voltage *U*_0_ applied between the two electrodes exceeds a critical threshold value *U*_c_ that corresponds to a critical strength of the defect. For lower voltages—that is, for weaker defects, it passes (see Fig. 1 and Supplementary movie 1).

Similarly in the case of conventional chemical or topographic surface patterns, the drop ‘feels’ the presence of the defect only when it overlaps with it. The (free) electrical energy of the system as a function of the drop position is *E*_el_(*x*)=−*c AU*^2^*ϕ*(*x*)/2, where *c* is the capacitance per unit area between the drop and the electrode(s) on the substrate, *A* is the area of the drop–substrate interface and *ϕ*(*x*) is a symmetric function varying smoothly between 0 for |*x*|>*R* and 0.25 at *x*=0 (see Methods section and Mannetje *et al*.^21^).

Including gravity, we obtain the net energy landscape experienced by the drop


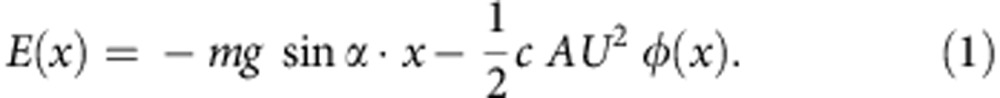


This energy landscape can be gradually tuned from a monotonically decreasing slope at zero voltage to a function with a well-defined local minimum at higher voltage, as illustrated in Fig. 1c.

**Figure 2:**
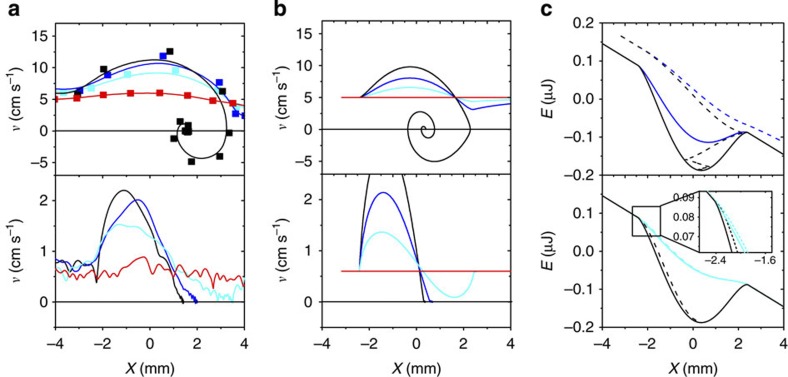
Drop velocity and energy versus drop position. Drop velocity ((**a**) experimental and (**b**) numerical) and drop energy (**c**) versus drop position upon passing a defect at variable voltage. Top row: pure water. Bottom row: water–glycerol mixture. Drop volume: 40 μl, inclination angle 5.3°. Applied voltage: *U*_0_=0 V (red), 200 V (turquoise), 300 V (blue) and 400 V (black). (**c**) Energy landscape (solid) and total energy (dashed) for selected voltages just below and above the critical trapping voltage. The kinetic energy is important for water but remains small at all times for the more viscous water–glycerol drops.

When the sliding drop reaches the wetting defect, it first accelerates because it is pulled into the potential well (see Fig. 2). As expected, higher voltages lead to faster acceleration. Furthermore, the absolute velocities at the same inclination angle are higher for water drops than for more viscous water–glycerol mixtures. After passing the centre of the trap, the drops slow down again. For low voltages, they escape the trap again at a final velocity close to the initial one and continue to slide downhill. Beyond the threshold voltage, however, the drops get trapped. In the case of water–glycerol drops, the speed of trapped drops gradually decreases from its maximum value to zero as the drops come to rest. In contrast, water drops oscillate a few times around their final equilibrium position before they eventually come to rest.

### Critical trapping conditions

**Figure 3:**
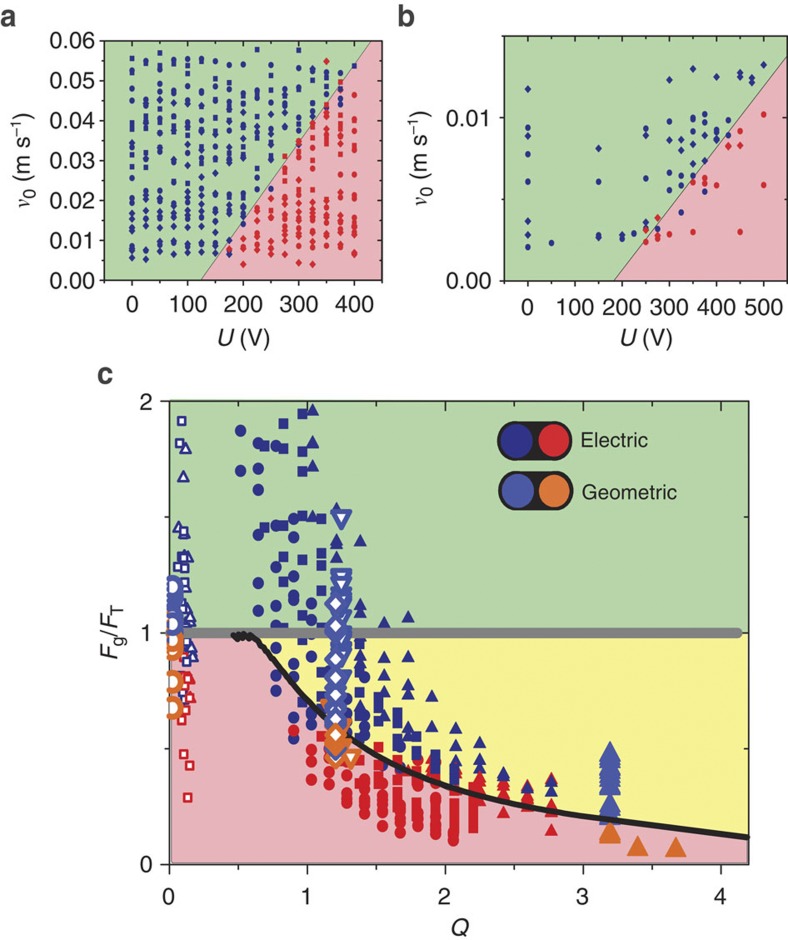
Trapping diagram at wetting defect. Red and blue symbols and coloured regions indicate trapped and passing drops, respectively. (**a**,**b**) Electrical trapping for drops of variable size (diamond: 20 μl; circle: 40 μl; square: 60 μl) as a function of initial velocity *v*_0_ and applied voltage *U*. (**a**) Pure water; (**b**) water–glycerol mixture. (**c**) Trapping diagram as a function of normalized driving force *F*_g_*/F*_T_ and quality factor *Q* of the trap (cf. equation 2). Green-shaded region: no potential minimum; yellow: potential minimum but escape by inertial overshoot; red: trapping. Horizontal grey line: quasistatic trapping limit in the absence of inertia. Solid black line: trapping limit in the presence of inertia. Small symbols: same data as in (**a**,**b**). Large symbols: superhydrophobic surfaces with mechanical defect. Data for electrical trapping are corrected for contact angle hysteresis reduction due to electrowetting^33^. See also Supplementary movie 3.

To determine the critical conditions for the trapping of drops, we repeated our experiments for various inclination angles and drop sizes with drops of both pure water and water–glycerol mixtures. For each condition, we determined the critical voltage *U*_c_ required for trapping. As expected, *U*_c_ is found to increase both with increasing inclination angle *α* and with increasing drop size. For the same drop size and inclination angle, we also find that water drops consistently require higher voltages—that is, stronger defects to become trapped than water–glycerol drops, despite the fact that the energy landscape is the same in both cases (up to a minor difference in density of <10%). Figure 3a, b shows a representation of all results as a function of the initial sliding velocity *v*_0_ and the trap strength measured in units of the applied voltage *U* for water and for water–glycerol drops. Blue symbols denoting drops that pass the trap are grouped at high initial velocities and low voltages. The red symbols denoting drops that get trapped are grouped at low initial velocities and high voltages.

Plotting the data as a function of *v*_0_ provides a more consistent representation than using the (sine of) *α*. At first glance, this may seem surprising since one would expect that the balance of the gravitational driving force *F*_g_=*mg* sin*α* and a viscous dissipation force *F*_d_=2*Rλv*_0_ (*R*: base radius of the drop; *λ*: viscous friction coefficient) leads to *v*_0_∝sin*α*. However, calibration measurements indicate a substantial influence of the small but finite pinning forces *F*_p_ due to the intrinsic roughness and contact angle hysteresis (see Methods section). These forces oppose sliding and lead to a reduced effective gravitational force *F*_g_=*mg* sin*α*–*F*_p_ (ref. 11). Irregularities due to random variations of *F*_p_ are automatically taken into account when representing the data as a function of the measured values of *v*_0_ rather than sin*α*. Next to a characterization of *F*_p_, the calibration measurements also yield quantitative values for the dissipation coefficients *λ*_w_=(4.5...6)·kg s^−1^ and *λ*_wg_=(1.2...1.3) kg s^−1^ for water and water–glycerol mixtures, respectively. In agreement with earlier findings^22^, these values suggest that the dissipation is governed by contact line friction rather than bulk dissipation. For the present situation of a lubricated drop on a surface with finite hysteresis, the contact line friction results from a combination of steady viscous dissipation^23^ and microscopic unsteady motion related to contact angle hysteresis^22^,^24^ (see Methods section).

To trap a drop, the potential well created by the defect needs to be sufficiently deep to generate a local minimum in the energy landscape rather than just a small depression (cf. Fig. 1c). In other words, the maximum of the trapping force *F*_t_ must be at least as strong as *F*_g_. In Fig. 3c, where the data of Fig. 3a,b are replotted as full and open small symbols, respectively; this means that all drops falling below the line *F*_g_/*F*_t_=1 (grey thick line in Fig. 3c) should become trapped and all drops above the line should pass. While this criterion is fulfilled (up to minor experimental uncertainties) for the water–glycerol drops, it dramatically overestimates the trapping capability of the same defect for pure water drops.

To understand the origin of this discrepancy, we describe the sliding drop as a point mass moving in the potential landscape generated by gravity and the wetting defect. Neglecting the internal degrees of freedom related to shape deformation of the drop may lead to inaccuracies regarding the details of the transient motion as the drop is being captured, yet, as we will see, the approach nevertheless captures the essence of the transition between weakly and highly viscous liquids.

To simplify our analysis, we approximate the electrostatic energy given in equation (1) by a generic harmonic potential *E*_el_(*x*)≈*k*(*U*) *x*^2^/2 (truncated at |*x*|=R) with a voltage-dependent stiffness *k*(*U*)=4 *cU*^2^/π (ref. 21). The maximum trapping force is then *F*_t_=*kR∝U*^2^*R*. For |*x*|>*R*, the drop does not feel the presence of the defect and hence the trapping force vanishes. With this approximation, Newton’s equation of motion for the centre of mass of the drop reads 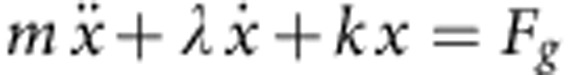 for |*x*|<*R*. *m* is the mass of the drop. This problem contains two characteristic time scales. First, the elasto-inertial time *T*_0_=2*π/ω*_0_ that is related to the eigen frequency 
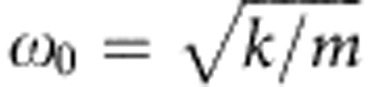
 of the drop in the trap. Second, the viscous relaxation time *τ*=2*m*/λ. (At first glance, one might suspect that there should be a third time scale given by the passage time *t*_0_=2*R/v*_0_ of an undisturbed drop past the defect. However, the specific initial conditions of a steadily sliding drop links v_0_ to τ. Hence, *t*_0_ is not independent.) Using *τ* as the unit of time and *R* as the characteristic length, we can rewrite Newton’s equation of motion in nondimensional form as


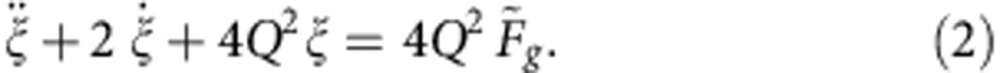


Here *Q=ω*_0_τ/2=*πτ*/*T*_0_ is the quality factor of our effective harmonic oscillator, and 
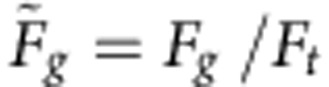
 is the normalized effective driving force. (Obviously, this quantity can also be regarded as an inverse normalized trapping strength 
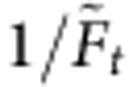
.) The ratio between trapping strength and driving force and the ratio between the viscous and the elasto-inertial time scale are thus the two dimensionless quantities that govern the trapping of sliding drops by wetting defects. Hence, *Q* is the second natural parameter next to 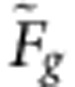 that we can use to represent all trapping measurements in a single graph (Fig. 3c).

The relevant criteria and different regimes of drop trapping are easily extracted from equation 2. First of all, for the drop to become trapped, there must be a stationary solution with vanishing drop speed and acceleration. The result is 
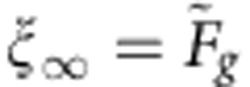
. Since *ξ* is constrained to values less than unity, 
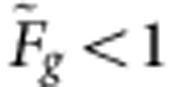
 immediately emerges as a necessary criterion for drops to become trapped, as expected. Indeed, all trapped drops in Fig. 3c fulfill this condition. Secondly, there are two different dynamic regimes, overdamped motion for *Q*<1 and underdamped motion for *Q*>1. In our experiments, the water/glycerol and the water drops represent these two different regimes: *T*_0_ ranges from about 0.1 s to 0.5 s for both types of drops, depending on the defect strength. From the values of *λ*, we find *τ*_wg_≈0.01*s*<<*T*_0_ and τ_w_≈0.2*s*=*O*(*T*_0_) for water–glycerol and water drops, respectively.

In the overdamped regime, visco-inertial relaxation takes place on much shorter times than the motion of the drop across the defect. Hence, inertia is irrelevant and the drop always moves with its momentary steady sliding velocity corresponding to the local slope of the energy landscape. As soon as the drop reaches the local energy minimum of the defect, it stops. This is illustrated by the calculated drop trajectories and the energy diagrams in the bottom panels of Fig. 2b,c. Note also that the total energy curve closely follows the potential energy, implying that the kinetic energy remains small at all times.

In the case of weak damping, on the other hand, *τ* becomes larger than *T*_0_. As a consequence, the drop dissipates little energy during a single passage of the defect: the kinetic energy becomes substantial. Even in the presence of a well-defined energy, minimum the drop can escape the defect with the help of inertia if the trap is too shallow. Only if the trap is deep enough, the viscous dissipation becomes strong enough to prevent drop escape. In this case, the drop oscillates a few times before coming to rest, as shown in the top panels of Fig. 2. The trapping criterion for *Q*>1 is given by the requirement that the drop actually stops and reverses direction before leaving the trap—that is, that 
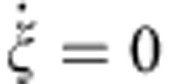
 is reached while *ξ*=*x*/*R*<1. Solving the corresponding analytical expressions numerically, we obtain the solid black line in Fig. 3c as a critical trapping condition. For *Q*<<1, this criterion approaches 
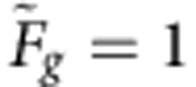
, as required. For *Q*>1, the critical value of 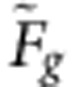 decreases and increasingly deeper defects are required to trap the drops, in agreement with our experimental observations.

## Discussion

The analysis presented above is rather generic. For any small wetting defect, the lateral extent of the drop–defect interaction is determined by the width of the drop. For a hydrophilic stripe of width *w*<<*R*, for instance, the depth of the potential well is given by Δ*E*=γ2*Rw*Δcos*θ*_*Y*_, where Δcos*θ*_*Y*_ describes the difference in wettability between the wetting defect and the homogeneous part of the surface. Knowing both depth and width of the wetting defect, we can approximate the trapping potential by a generic harmonic potential *E*_trap_(*x*)≈*kx*^2^/2, where *k*≈4γ*w*Δcos*θ*_*Y*_/*R* for a chemical stripe defect. Like in the case of electrical defects, details of the potential shape should not affect the generic scenario as long as contact angle hysteresis and sliding speeds are not too high, such that macroscopic drop deformations during sliding are prevented. Many novel functional surfaces such as superhydrophobic, superoleophobic and oil-impregnated nanotextured^25^,^26^,^27^ surfaces fulfill these criteria. To test the general applicability of our approach, we fabricated nanotextured polymeric superhydrophobic surfaces with *θ*_*Y*_=160°and Δ*θ*≈3°. These surfaces were scratched with a sharp needle under controlled load, resulting in a combined topographic and chemical defect due to local removal of the hydrophobic polymer layer. The trapping force of these defects is determined experimentally from the critical inclination angle at which an already pinned drop is released. The large symbols in Fig. 3c show the results for a series of drops of various water–glycerol mixing ratios. Similarly for the electrical defects, very viscous drops with *Q*<<1 get trapped whenever 
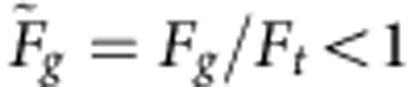
. Less viscous drops can also escape for smaller values of 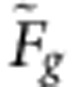. The transition between trapping and non-trapping follows the critical condition predicted by our model (see Fig. 3c).

**Figure 4:**
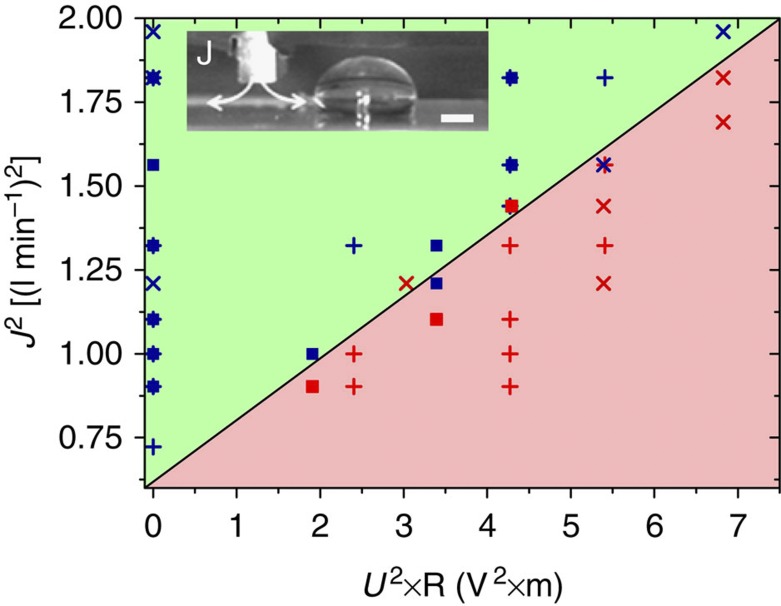
Trapping diagram for different air flow rate. Trapping diagram for sessile water–glycerol drops of variable size (square: 20 μl; plus: 40 μl; cross: 80 μl) driven by an air jet with volumetric flow rate *J*. Transition between trapping (red) and passing (blue) reflects the force balance between driving air drag and electrical trapping forces. Inset: experimental geometry. The electrical defect is located ~5 mm to the right of the slot-like nozzle. Scale bar: 1 mm.

The same concept can also be applied to other driving forces than gravity such as viscous drag by ambient flows of air or of a second immiscible fluid. Figure 4 shows an example of an air jet impinging on a solid surface, as used, for example, in cleaning applications to remove drops from surfaces. Under these conditions, the drag force scales with the square of the volumetric air flux *J*^28^. Consequently, the critical pinning condition of glycerol–water drops on a surface with an electrical defect follows the expected scaling 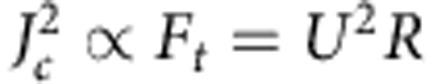 (Fig. 4). Experiments in oil–water two-phase flow microfluidic devices fall into the purely viscous regime due to the damping caused by the ambient oil and display an excellent agreement between the critical trapping and the viscous drag forces^29^.

**Figure 5:**
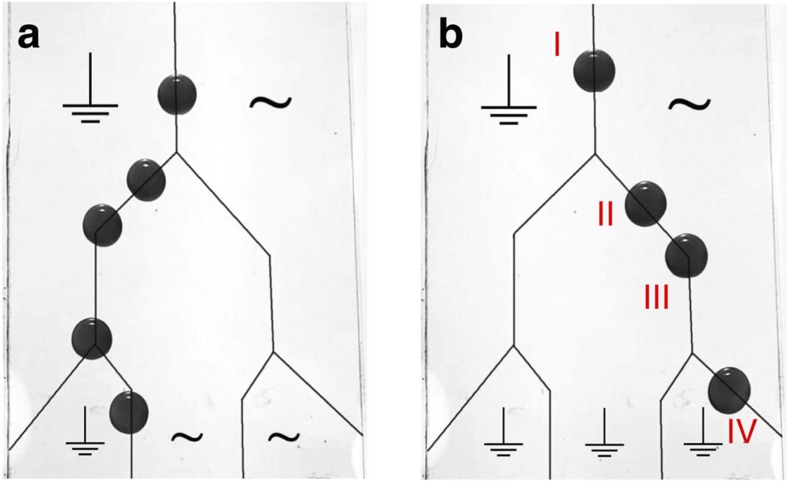
Top view of drop steering by electrically switchable guides on inclined plane. (**a**) and (**b**) indicates the drop steering at different directions by actuating the voltage accordingly. Symbols indicate actuation state–that is, grounded versus applied voltage for each of the five electrodes. At each Y-junction, drops follow the track determined by the applied voltage as indicated by the arrows. Drops slide at steady-state sliding velocity along sections I and III and slow down on inclined sections II and IV due to the reduced effective driving force. See also Supplementary movie 2.

Finally, we want to point out that the trapping principle described above can also be applied to steer drops along certain directions on the surface. Figure 5 illustrates the principle for linear electrical defects oriented at an inclination angle of 45° with respect to the sliding direction. While previous attempts using surfaces with inclined stripes of a different chemical nature^30^ and roughness and contact angle hysteresis^31^ displayed a lateral displacement of passing sliding drops, we can increase the strength of our electrical defects to capture the drops and guide them along the path prescribed by the electrode pattern. Once captured by the defect, the drop moves along the defect under the influence of the parallel component of the driving force. For the electrically addressable defects used here, arbitrary geometries of defects and guides are readily designed and allow for steering drops in desired directions, as shown in Fig. 5 (see Supplementary movie 2). We anticipate that the principles described here will enable flexible drop control in various applications including sorting based on drop size.

## Methods

### Sample preparation

The substrates for the experiments with electrical defects consist of glass plates covered by a 30-nm-thin transparent electrode of indium tin oxide. HCl is used to etch a gap of 0.5 mm width into the indium tin oxide layer to generate two separate electrodes forming the electric trap. Adhesive tape (Scotch Pressure Sensitive)—a polypropylene film with a nominal thickness of 28–52 μm (including glue)—is used as an insulating layer to cover the electrodes. A thin layer of silicone oil (viscosity 5 mPas) is applied to the surface resulting in advancing and receding contact angles of 95° and 92°, respectively. The effective dielectric thickness of the substrate is *d*≈40 μm, as determined from the electrowetting response of the system assuming a dielectric constant of *ε*=2. Alternating voltages (AC) of up to 500 V root-mean-square at a frequency of 1 kHz are applied to generate the electric traps resulting in a contact angle for trapped drops of (78±3)° at the highest voltage. KCl was dissolved in deionized water and 1:8 (vol-vol; nominal viscosity: 56 mPas) water–glycerol mixtures to guarantee a conductivity of ~3 mS cm^−1^.

Nanostructured superhydrophobic surfaces are prepared by a protocol adapted from Gnanappa *et al*.^32^. Thin films of SU8 photoresist are first exposed to an oxygen plasma to generate nanoscale roughness and subsequently hydrophobized by depositing a fluorinated top coating generated from a C_4_F_8_ precursor.

### Electric trapping

When the conductive water drop reaches the gap, it forms two capacitors *C*_1_(x) and *C*_2_(x) with the two electrodes on the substrate. Both capacitances depend on the geometric overlap A_i_(x) of the drop with electrode *i*: *C*_*i*_(*x*)=*cA*_*i*_(*x*) (*i*=1,2) where *c*=εε_0_/*d* is the capacitance per unit area between the drop and the electrodes. Neglecting the finite width of the gap between the electrodes, the resulting net capacitance of the system (see effective circuit model in Fig. 1b) is *C*(*x*)=*C*_1_(*x*) *C*_2_(*x*)/(*C*_1_(*x*)+*C*_2_(*x*))=*cAϕ*(*x*), where 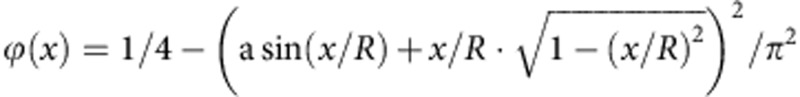. Note that the electrically isolated drop chooses its potential between the potential of the two electrodes according to its position. For *x*=0, the voltage between the drop at the electrodes is *U*/2.

### Calibration of dissipation coefficient

**Figure 6:**
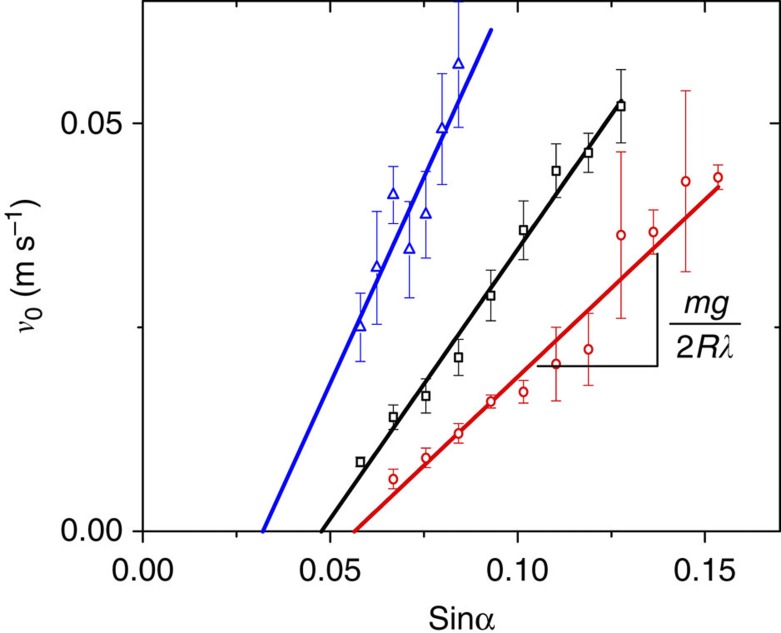
Steady-state sliding velocity versus sine of the inclination angle. Red, black and blue data points are the drop velocities at different inclination angle for 20, 40 and 60 μl drops respectively. Straight lines are the linear fits.

To determine the damping coefficient of the sliding drops on the homogeneous surface, we measured the steady sliding velocity *v*_0_ away from the trap as a function of inclination angle. Figure 6 shows the expected linear relation between *v*_0_ and sin*α*. Owing to the finite residual hysteresis, the curves intercept the abscissa at a finite value sin*α*_0_. The balance between gravity, viscous friction and pinning is


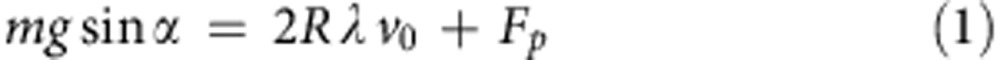


we can determine both λ and *F*_p_ from the these calibration measurements. The pinning forces are related to contact angle hysteresis by *F*_p_=2*R*γ(cos*θ*_*r*_–cos*θ*_*a*_), where *θ*_r_ and *θ*_a_ are the receding and the advancing contact angle, respectively. From this expression, we find sin*α*_0_=2*R*γ(cos*θ*_r_–cos*θ*_a_)/*mg*, leading to a critical sliding angle *α*_0_=1…3°, decreasing with increasing drop size, in agreement with the experimental results (Fig. 6).

From the slope of the calibration curves in Fig. 6, we can extract the damping coefficients *λ*_w_=(4.5±0.2) × 10^−4^, (5.9±0.1) × 10^−4^, (5.8±0.5) × 10^−4^ kg s^−1^ for the water drops of 20, 40 and 60 μl, and *λ*_wg_=(1.22±0.03) × 10^−2^ kg s^−1^ and (1.32±0.03) × 10^−2^ kg s^−1^ for the 40 and 60 μl water–glycerol drops. This weak size dependence is attributed to the different scaling of driving, viscous and pinning forces with the drop size. For the purpose of our analysis, this minor variation can be neglected. Dividing the values of *λ* by the drop width 2*R*, we find values for the contact line friction coefficient *ξ*_*w*_≈100 *mPa·s* and *ξ*_*wg*_≈5 *Pa·s*. The value for water is somewhat larger than the one extracted in earlier experiments with sliding drops on dry substrates^22^. The additional contribution may arise from the viscous dissipation of the oil film on the substrate as described by Smith *et al*.^23^

## Author contributions

D.M., A.P., S.G., R.L. and S.O. carried out the inclined plane experiments. D.M., C.B. and J.Z. designed and executed the air jet experiments. D.M. and D. vdE. analysed the experimental data. F.M. designed the research. D.M. and F.M. wrote the manuscript.

## Additional information

**How to cite this article:** 't Mannetje, D. *et al*. Trapping of drops by wetting defects. *Nat. Commun.* 5:3559 doi: 10.1038/ncomms4559 (2014).

## Supplementary Material

Supplementary Movie 1 video sequence showing two water drops sliding down and inclined plane for applied voltages below (left) and above (right) the critical voltage.

Supplementary Movie 2 video sequence of drops sliding down an inclined plane with individually addressable electrodes to guide the drops at junctions. Each time a drop has passed, one additional electrode is activated. As a consequence subsequent drop is directed along a different path. Cf. Fig. 5.

Supplementary Movie 3 Video sequence showing next to each other two drops sliding down after each other the same inclined plane with a superhydrophobic surface with a scratch. For the first drop the inclination angle (a) is 15° and the drop gets trapped. For the second drop, a=19° and the drop passes the trap.
